# Characterization of the karyotype and accumulation of repetitive sequences in Australian Darling hardyhead *Craterocephalus amniculus* (Atheriniformes, Teleostei)

**DOI:** 10.7717/peerj.7347

**Published:** 2019-07-30

**Authors:** Zuzana Majtánová, Karl G. Moy, Peter J. Unmack, Petr Ráb, Tariq Ezaz

**Affiliations:** 1 Laboratory of Fish Genetics, Institute of Animal Physiology and Genetics, Czech Academy of Sciences, Libechov, Czech Republic; 2 Institute for Applied Ecology, University of Canberra, Canberra, Australian Capital Territory, Australia

**Keywords:** Fish cytotaxonomy, Karyotype, FISH, rDNA, Chromosomal markers, Atherinidae, Heterochromatin, Telomeres, Chromosomes

## Abstract

Belonging to the order Atheriniformes, *Craterocephalus* is one of the most widespread genera of freshwater fishes in Australia, spanning along the northern coast from central Western Australia to central New South Wales and across the Murray-Darling and Lake Eyre basins. In this study, both conventional cytogenetic techniques (Giemsa, C-banding, CMA_3_/DAPI staining), and fluorescence *in situ* hybridization (FISH) with telomeric DNA and rDNA probes were used to examine the karyotypes and other chromosomal characteristics of Darling hardyhead (*Craterocephalus amniculus*) from New South Wales, Australia. We identified a diploid chromosome number 2*n* = 48 (*NF* = 58) in all studied individuals. FISH with rDNA probes showed a nonsyntenic pattern, with signals on one pair of subtelocentric chromosomes for 5S rDNA and one pair of submetacentric chromosomes for 28S rDNA. C-banding displayed the accumulation of constitutive heterochromatin in the centromeric regions of approximately 40 chromosomes. CMA_3_/DAPI fluorescence staining revealed extremely GC-rich signals in the pericentromeric region of one submetacentric chromosomal pair with size polymorphism. We detected telomeric signals at the end of all chromosomes and no interstitial signals.

## Introduction

Atherinidae, commonly known as Old World silversides, is a widespread family found primarily in marine and estuarine waters throughout the Atlantic and Indo-West Pacific (Nelson, Grande & Wilson, 2016). This family is comprised of four subfamilies, 14 genera, and 75 species (Fricke, Eschmeyer & Van der Laan, 2019). *Craterocephalus* is the type genus for the subfamily *Craterocephalinae* which it shares only with the genus *Sashatherina* (Dyer & Chernoff, 1996; Ivantsoff & Allen, 2011; Campanella et al., 2015). It is one of the few genera within the family which have undergone an extensive freshwater radiation, with most species restricted to Australia, New Guinea and Timor-Leste, although a small number inhabit marine environments in Australia (Unmack & Dowling, 2010). Moreover, *Craterocephalus* is one of the most widespread genera of freshwater fishes in Australia, spanning the northern coast from central Western Australia to central New South Wales and across the Murray-Darling and Lake Eyre basins (Unmack & Dowling, 2010). Inherent to such a widespread distribution, members of this genus inhabit a broad range of habitats including arid intermittent rivers, desert springs and lakes, through to large perennial rivers, smaller creeks and marine mangroves, beaches and estuarine areas (Allen, Midgley & Allen, 2002).

The Darling hardyhead, *C. amniculus* is a small bodied species with an annual lifecycle and a habitat preference for deep slow flowing pools (Moy, Wilson & Ellison, 2018). This species is naturally distributed throughout the upper tributaries of the Darling River of the Murray-Darling Basin and the coastal Hunter River (Adams et al., 2011). Recently, the species has declined across parts of its range and is thought to be under threat from habitat loss and degradation (Williamson, 2014), though it is often abundant in areas which it is still present. Within *Craterocephalus, C. amniculus* belongs to the ‘eyresii’ clade and is the sister taxon to *C. fluviatilis* (Unmack & Dowling, 2010).

Despite the fact that numerous studies regarding the taxonomy and phylogeny of the genus have been published (Crowley & Ivantsoff, 1990; Crowley & Ivantsoff, 1992; Unmack & Dowling, 2010; Adams et al., 2011), no comparative cytogenetic research has been performed. So far only two atherinid species have been studied cytogenetically (Vasil’ev, 1980; Kikuno & Ojima, 1987), neither of which are from Australia. In this report, we characterized for the first time, the karyotypes of *C. amniculus* using a set of conventional as well as molecular cytogenetic tools. We describe the chromosomal number, together with the numbers and positions of minor and major rDNA genes, heterochromatin and telomeric sequences. This study thus extends the knowledge about the genome evolution of this diverse genus.

## Material and Methods

### Specimens

Twenty-five juvenile individuals were cytogenetically analysed in this study. Parents of analysed individuals were collected from Swan Brook in the McIntyre River (29°46′18.68″S 151°26′7.11″E) in March 2018 using a seine net under the NSW Fisheries Permit No: P07/0007-5.0. These fish were bred in captivity with the offspring raised in separate aquariums. All samples were obtained under state fisheries permits and research was conducted with approval from the University of Canberra ethics committee (CEAE.15-05).

### Chromosome preparation and staining

Chromosomal suspensions were obtained from four week old juvenile individuals using the protocol described by Netto, Pauls & Affonso (2007) with slight modifications. Fish were euthanized using an overdose of anaesthetic (clove oil). Whole fish were homogenised to produce the cell suspension and cultured for 10 hours in RPMI 1640 medium (Life Technologies, Inc., Carlsbad, CA, USA) at 4 °C. Colchicine treatment was performed for one hour, followed by hypotonization in 0.075 M KCl for 50 min at room temperature. Cell suspensions were fixed in freshly prepared fixative (methanol: acetic acid 3:1, v/v), washed twice in fixative, and finally spread onto slides. Chromosomal preparations were stained with Giemsa solution (5%, 10 min) to identify the number and morphology of chromosomes in all 25 individuals used in this study. C-banding, visualizing blocks of constitutive heterochromatin was performed according to Sumner (1972) with slight modifications as described in Pokorná et al. (2014). After C-banding the chromosomes were counterstained with Vectashield DAPI anti-fade medium (Vector Laboratories, Burlingame, CA, USA) to enhance the contrast, and the microphotographs were taken in the fluorescent regime and inverted. Chromomycin A_3_ (CMA_3_) staining was performed to reveal GC genome composition as described by Sola et al. (1992) using Vectashield DAPI anti-fade medium (Vector Laboratories, Burlingame, CA, USA) as a mounting reagent.

### Fluorescence *in situ* hybridization with telomeric probe and rDNA genes

The topology on the karyotype of the telomeric motif (TTAGGG)_n_ and the rDNA genes within the genomes were analysed by fluorescence *in situ* hybridization (FISH). Telomeric FISH was performed using a Cy3-labelled PNA probe according to the manufacturer’s instructions (Telomere PNA FISH Kit/Cy3, Dako). Probes for rDNA FISH experiments were produced by PCR with the following primer pairs: (a) 5S rDNA: 5′-TACGCCCGATCTCGTCCGATC-3′ and 5′-CAGGCTGGTATGGCCGTAAGC-3′(Komiya & Takemura, 1979); (b) 28S rDNA: 5′-AAACTCTGGTGGAGGTCCGT-3′ and 5′-CTTACCAAAAGTGGCCCACTA-3′(Zhang, Cooper & Tiersch, 2000). The PCR reactions were carried out in a final volume of 25 µL consisting of 100 ng genomic DNA, 12.5 µL PPP master mix, 0.01 mM of each primer and PCR water to complete the volume (all reagents from TopBio, Prague, Czech Republic). Cycling conditions were as follows: (a) 5S rDNA: 5 min at 94 °C; 35 cycles of 45 s at 94 °C, 30 s at 55 °C and 45 s at 72 °C; 10 min at 72 °C; (b) 28S rDNA: 5 min at 94 °C; 35 cycles of 45 s at 94 °C, 30 s at 53 °C and 45 s at 72 °C; 10 min at 72 °C. Probes were indirectly labelled with biotin-16-dUTP (Roche, Mannheim, Germany) and digoxigenin-11-dUTP (Roche) through PCR reamplification of PCR products. Reamplification was carried out under the same conditions as the previous PCR reaction. Labelled PCR products were precipitated. A hybridization mixture was made consisting of hybridization buffer (Symonová et al., 2015), salmon sperm blocking DNA (15 µg/slide; Sigma-Aldrich, St. Louis, MO, USA) and differently labelled PCR products of both genes. The hybridization and detection procedures were carried out under conditions described by Symonová et al. (2015). The biotin-dUTP-labelled probes were detected by Invitrogen Cy™3-Streptavidin (Invitrogen, San Diego, CA, USA; cat. no. 43-4315), the digoxigenin-dUTP-labeled probes were detected by Anti-Digoxigenin-Rhodamin (cat. no. 11207750910). Finally, the slides were mounted with Vectashield DAPI anti-fade medium (Vector Laboratories, Burlingame, CA, USA).

### Microscopy and image analyses

Chromosomal preparations were examined by an Olympus Provis AX 70 epifluorescence microscope. Images of metaphase chromosomes were recorded with a cooled Olympus DP30BW CCD camera. The IKAROS and ISIS imaging programs (Metasystems, Altlussheim, Germany) were used to analyse grey-scale images. The captured digital images from FISH experiments were pseudocolored (red for Anti-Digoxigenin-Rhodamin, green for Invitrogen FITC-Streptavidin) and superimposed using Adobe Photoshop software, version CS5. For CMA_3_/DAPI staining, the CMA_3_ signal was inserted into the red and the DAPI signal into the green channel to enhance the contrast between these two types of signals. The chromosomal categories were classified according to Levan, Fredga & Sandberg (1964) and chromosomes were ordered in decreasing size order.

## Results

**Figure 1:**
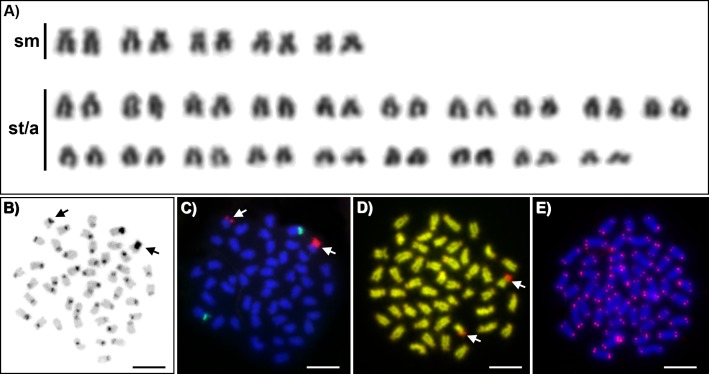
Cytogenetics of *Craterocephalus amniculus*. (A) Representative karyotype arranged from Giemsa-stained chromosomes. (B) C-banded chromosomes. The C-banding pattern was counterstained with DAPI and inverted; (C) Metaphase chromosomes after FISH with 5S rDNA (green signals) and 28S rDNA (red signals) probes; (D) CMA_3_/DAPI stained chromosomes. The CMA_3_ signal was converted from green into red and the DAPI signal from blue into green to enhance the contrast between these signals. (E) Metaphase chromosomes after FISH with a telomeric probe. Particular images were adjusted separately before assembling the plate. Arrows indicate the pair of homologous chromosomes with the accumulation of heterochromatin. Bars equal 10 µm.

Karyotypes of all studied individuals possessed the same diploid chromosomal number, 2*n* = 48. Fundamental number (number of chromosomal arms) NF = 58 (Fig. 1A). At least five metaphases per individual have been inspected. A subset of 10 individuals with the most suitable chromosomal spreads was selected for further chromosomal analyses. C-banding revealed the accumulation of constitutive heterochromatin in the centromeric regions of approximately 40 chromosomes (Fig. 1B). One submetacentric chromosome possessed accumulation of heterochromatin on the whole p arm. FISH with an rDNA probe showed a nonsyntenic pattern. FISH signals were detected in one pair of subtelocentric chromosomes for 5S rDNA and one pair of submetacentric chromosomes for 28S rDNA (Fig. 1C). CMA_3_/DAPI fluorescence staining revealed mostly homogeneously stained chromosomes with a balanced proportion of AT/GC, moderately GC-rich centromeric regions and extremely GC-rich signals in the centromeric region of one submetacentric chromosomal pair with size polymorphism in all individuals (Fig. 1D). We detected telomeric signals at the end of all chromosomes but no interstitial telomeric sites (ITSs) in studied individuals (Fig. 1E) were presented. Notably, the p-arm of a single chromosome in all metaphases of all studied individuals showed C-positive heterochromatin accumulation (Fig. 1B), extensive amplification of 28S rDNA loci (Fig. 1C) and GC-rich content after CMA_3_/DAPI staining (Fig. 1D).

## Discussion

Genomes of teleost fishes have intrinsic characteristics that may be involved in the formation of the amazing diversity of this group. Particularly, there is now substantial evidence that an ancient event of genome duplication (tetraploidization) has provided the evolutionary framework for the diversification of gene functions and for extensive speciation events in teleost fishes (Volff, 2005). Karyotypes of several thousand fish species have been described in the decades since the development of various methods for characterizing chromosome sets (for the last comprehensive review see Arai (2011)), providing valuable information about the structure and evolution of fish genomes. However, similarly to other fish families in Australia, representatives of the family Atherinidae have been omitted from even basic conventional cytogenetic analyses. Two previously studied Atherinidae species, *Atherina mochon* and *Hypoatherina bleekeri*, demonstrated diploid chromosome numbers 2*n* = 48. In this study we provide the first karyotype description of *C. amniculus* which is distributed throughout the Darling River system in Australia. The diploid chromosomal number of *C. amniculus* is 2*n* = 48, which is in concordance with previously published results, implicating karyotype stability in the speciation of this clade. Nevertheless, previously studied species differ in NF; karyotype of *Atherina mochon* (= *Atherina boyeri*) display NF = 54 (Vasil’ev, 1980), *Hypoatherina bleekeri* (= *Hypoatherina valenciennei*) display NF = 96 (Kikuno & Ojima, 1987) and *C. amniculus* possess NF = 56. These differences suggest microstructural chromosome changes in centromeric positions during the evolutionary diversification process. Unfortunately, more detailed comparison of atherinid species examined as yet with *C. amniculus* is impossible due to the lack of published information. No differentiated sex chromosomes have been described in previous analyses of species belonging to this clade. Similarly, we did not observe any karyotypic differences among the 25 individuals studied. *Craterocephalus amniculus* displayed two signals for both major and minor rDNA genes located on nonsyntenic chromosomes (Fig. 1B). This pattern is the most frequent for the majority of teleost species (Gornung, 2013). 28S rDNA signals displayed a size heteromorphism between homologous chromosomes, which has also been observed in *Atherinella brasiliensis*, a related lineage in the Atheriniformes from the family Atherinopsidae (Sczepanski et al., 2007). 28S rDNA signals correspond to GC-rich signals revealed by CMA_3_/DAPI fluorescence staining (Fig. 1C) and also co-localizes with constitutive heterochromatin, which was overall located in the pericentromeric regions of 40 chromosomes. In order to document interstitial telomeric sites (ITSs) as remnants of chromosomal rearrangements, we employed FISH with conserved vertebrate telomeric repeat (TTAGGG)_n_. However no ancestral chromosomal fusions represented by ITSs were detected in *C. amniculus*.

In conclusion, our study revealed, for the first time, the karyotype and other chromosomal characteristics of *C. amniculus* and that this species has retained the karyotypic structure stability (2*n* = 48) typical for the family Atherinidae. By using advanced cytogenetic techniques, our results have extended the knowledge of genome organization in the family Artherinidae. Nevertheless, detailed cytogenetic characterization of more Atherinid species is needed to provide a comprehensive overview of comparative cytotaxonomy of the group.

## Additional Information and Declarations

### Field Study Permissions

The following information was supplied relating to field study approvals (i.e., approving body and any reference numbers): NSW Fisheries Permit No: P07/0007-5.0 allowed the use of a seine net for collection. No other fieldwork permissions were required.

### Data Availability

The following information was supplied regarding data availability: The raw data is available within the Results section and Fig. 1.
